# Evidence for Automatic, Stimulus Driven, Arithmetic Processing of Single-digit Multiplication Problems

**DOI:** 10.5334/joc.372

**Published:** 2024-06-05

**Authors:** Eldad Keha, Daria Klotsvog, Sarit Ashkenazi, Eyal Kalanthroff

**Affiliations:** 1 Department of Psychology, The Hebrew University of Jerusalem, Jerusalem, Israel; 2 Department of Psychology, Achva Academic College, Arugot, Israel; 3 The Seymour Fox School of Education, The Hebrew University of Jerusalem, Jerusalem, Israel

**Keywords:** Stimulus-driven behavior, automaticity, selective attention, numerical cognition, task conflict

## Abstract

Certain stimuli can automatically trigger different behaviors in a stimulus-driven manner. To investigate whether mathematical equations automatically trigger the tendency to engage in arithmetic processing, we asked whether the presentation of multiplication equations in an irrelevant dimension can trigger the automatic task of arithmetic processing and if so, which processes are involved. To that end, we employed a color-naming task in which participants had to name the color of different stimuli, such as: mathematical equations (e.g., 4 × 6 = 24), neutral-symbols (e.g., ####), neutral-words (e.g., building), and same-number strings (e.g., 11111), which appeared as one of four different colors. We found that mathematical equations and regular words in the irrelevant dimension triggered more task conflict (i.e., color naming’s reaction time was longer) as compared to same-number strings. In addition, we found evidence for the automatic activation of different numerical processes; such that large-size equations (7 × 9 = 63) triggered more conflict as compared with small-size (2 × 3 = 6) equations and same-parity incorrect equations (3 × 2 = 8) triggered more conflict as compared to different-parity incorrect equations (4 × 2 = 9). We found no evidence indicating a distinction between the correct and incorrect equations. We discussed the relevance of the findings to the automaticity of arithmetic abilities and other domains in numerical cognition.

Some aspects of numerical cognition are considered to be automatic in the sense that they do not require intentional processing (Tzelgov et al., 1992). For example, a very robust finding in the literature on numerical cognition is that the numerical value of a number affects the perception of its physical size (Henik & Tzelgov, 1982; Szűcs & Soltész, 2007). These evidence suggest that processing numerical information is a primary and automatic function. Other classic theories in the field of numerical cognition suggest that different mental processes such as arithmetic operations are automatic (Logan, 1988) and that arithmetic calculations are based on a direct-access, single-step retrieval of solutions from memory rather than some algorithmic computation. Logan, in his ‘instance theory’ (Logan, 1988) suggested that learning arithmetic operations, such as solving multiplication equations, is a fast, automatic, and obligatory process of retrieving practiced instances of the correct answer from memory. In the present study, we aim to investigate the automaticity of math equations (specifically through the task of color naming of various multiplication equations) and to ask whether the mere presence of these equations in an irrelevant dimension is sufficient to trigger different mental arithmetic processes, and in case it does, what kind of arithmetic processes are involved.

As proposed by Monsell (2003), some stimuli are not only processed automatically but can also be strongly associated with a particular task. For example, according to Gibson’s (1977) theory of affordances, common manipulatable objects trigger responses that have acquired a strong association with them. This results in automatic and specific motor plans for interacting with them. Such motor plans take place even in the absence of an explicit intention for interaction (Allport & Wylie, 2000; Makris et al., 2013), as is evidenced by the automatic activation of the pre-motor cortex when viewing manipulatable objects (Chao & Martin, 2000; Creem-Regehr & Lee, 2005; Proverbio et al., 2011). In addition, it has been shown that certain objects might trigger the tendency to name them (La Heij & Boelens, 2011) and that words automatically trigger the tendency to read them (Monsell, 2003; Perlman & Tzelgov, 2006). These examples suggest that both motor and mental behaviors can be activated in an automatic, stimulus-driven manner. Importantly, when these stimulus-driven behaviors are incongruent with one’s goal, a task conflict emerges between the relevant and the irrelevant stimulus-driven task (Littman et al., 2019; see Parris et al., 2023, for an opposing suggestion). In the current investigation, we ask whether arithmetic operations might trigger a stimulus-driven behavior similarly, and if so, what this behavior might be.

Mental arithmetic is a basic cognitive skill in human behavior and it involves several executive functions (Imbo & Vandierendonck, 2007; Lemaire, 1996). In both children and adults, solving simple equations (e.g., 2 × 6, 3 + 4) develops from an algorithmic strategy, in which participants use different calculation strategies to solve the equations, and gradually, with age and experience, advance towards a direct and fast retrieval of the correct answer from memory (Ashcraft, 1982; Logan, 1988; Siegler, 1988). In other words, with extensive practice, solving basic arithmetic equations becomes automatic. Specifically, it has been suggested that multiplication equations almost exclusively depend on fact retrieval from memory (Grabner et al., 2022). This notion has been supported by brain imaging studies which show neural activity that is involved in retrieving semantic knowledge from memory while engaging with multiplication equations (Chochon et al., 1999; Ischebeck et al., 2007; Jost et al., 2004; Zhou et al., 2007). Some of the studies that tested neuronal markers for multiplication equations, found a specific multiplication-related activity in the precentral gyrus and the supplementary motor areas and stronger activity for large equations (8 × 9 = 72) compared to small equations (2 × 5 = 10; Jost et al., 2004; Zhou et al., 2007). Researchers have argued that these neuronal differences between large and small-size equations indicate that different processes occur in small-size equations, which can be retrieved automatically, and large-size equations, which require algorithmic strategies.

Another process that has been found to take effect when engaging with multiplication equations is the processing of equations’ parity. A few studies have shown that participants can use information regarding the parity of the equation when performing mental arithmetic operations (Lemaire & Reder, 1999; Lochy et al., 2000). It is usually found in these studies that participants reject a false equation more rapidly when its parity is mismatched with the parity of the correct answer (e.g., 6 × 4 = 23) than when it is matched with it (e.g., 6 × 4 = 22). Taken together, different responses to small vs. large equations and to same vs. different-parity incorrect equations could suggest that arithmetic processing takes place. Thus, in the current study, we can use these markers to determine whether the stimulus-driven behavior triggered by simple mathematical equations (even when participants are not required to act on these equations) is indeed related to these arithmetical processes.

There is evidence in the literature that math equations are automatically processed when it is task-irrelevant (e.g., LeFevre et al., 1988; Galfano et al., 2003). These studies used the numbers-matching paradigm in which participants were presented with two single-digit cues (e.g., 3 7) which were followed by a two-digits probe (e.g., 18). The task was to decide whether one of the numbers in the cue also appeared in the probe. Galfano et al. found that participants were slower to respond when the probe was closer to the product of the two single-digits cues. For example, reaction time (RT) was slower when the cue was 3 7 and the probe was 18, compared to when the cue was 3 7 and the probe 12. Similarly, in an event-related potential (ERP) study that used the same paradigm, Galfano et al. (2004) reported stronger ERP negative activity at N400 amplitude and slower RTs when the probe was the correct result of multiplying the cue’s two single digits (e.g., cue: 3 7; probe: 21) compared to trials in which the probe was not (cue: 3 7; probe: 15). The authors concluded that participants retrieved the arithmetic information in an automatic stimulus-driven fashion. Taken together, these previous findings not only demonstrate a stimulus-driven behavioral process in handling irrelevant arithmetic information but also demonstrate the involvement of memory retrieval processes. The distinction between various equation types (large vs. small, same vs. different-parity, correct vs. incorrect) allows examination of these processes. Notably, previous studies mostly assessed these processes in the relevant dimension and the few studies that demonstrated automatic stimulus-driven arithmetic processing in the irrelevant dimension, utilized a relevant task that necessitated numerical processing.

Building upon the aforementioned findings, our present study aims to achieve two primary objectives. First, we aim to contribute to the existing literature by offering direct evidence that arithmetic information can be activated in a stimulus-driven manner, even during the execution of an entirely non-numerical relevant task. Second, if mathematical equations automatically trigger arithmetic processing, our second goal is to investigate the specific processes—previously observed to impact responses in the irrelevant dimension—that manifest when such arithmetic information emerges within this irrelevant context. As noted above, we will focus on multiplication equations. These types of equations are usually presented to participants either as a production task, in which participants are asked to generate the correct answer (e.g., 2 × 3 = ?), or as a verification task, in which participants are asked to decide whether the given equation is correct or not (e.g., 2 × 3 = 5). Logan (2002), following Krueger (1986), suggested that the verification task is based on plausibility and familiarity, since participants need to decide if the answer is plausible or not within a short period of time (commonly less than 1 second). Most importantly, the verification task does not necessarily require memory retrieval or generation of the correct answer (see also: Lemaire & Reder, 1999) and in most cases, participants report that they do not calculate the correct answer (Campbell & Fugelsang, 2001). Therefore, in our current study, we will employ a color-naming task more suitable for assessing irrelevant processes involving engagement with arithmetic information. This approach will enable us to investigate three distinct processes within the irrelevant dimension. Firstly, the comparison between large and small-sized equations will offer additional insights into whether participants, beyond the automatic processing of arithmetic information, are influenced by memory retrieval algorithmic processes characteristic of larger equations rather than solely by the complexity of the visual stimuli (De Smedt et al., 2011; Imbo & Vandierendonck, 2008; Núñez-Peña, 2008). Secondly, plausibility assessment will involve comparing incorrect equations with the same-parity (more plausible) vs. incorrect equations with different-parity (less plausible). Finally, exploring the role of equation familiarity in the irrelevant dimension will entail comparing correct (and more familiar) equations with incorrect equations.

To test all these processes, we administered a novel task, in which participants were asked to respond to the ink color of visual stimuli, including different math equations. Control conditions included: neutral-symbols (e.g., ####), which are known to trigger minimal (if any) task conflict; same-number strings (e.g., 111111), which as mentioned above, are expected to trigger task conflict to some extent, given that numbers might trigger a competing numerical processing task; and neutral-words (e.g., building), which are known to trigger the competing task of reading and hence trigger task conflict (Goldfarb & Henik, 2007). To tease apart the behavioral manifestation of task conflict, we used a low-control procedure with an increased number of neutral-symbols, which is known to reduce control levels and increase the behavioral manifestation of stimulus-driven behavior (Kalanthroff et al., 2018; Keha & Kalanthroff, 2023; Littman et al., 2019).

Our initial hypothesis posited that regardless of equation type when presented in the irrelevant dimension, math equations would be automatically processed and induce greater task conflict than number strings. We anticipated that RTs for math equations would be longer compared to the neutral symbols condition and even compared to the same-number strings condition. Regarding the distinction between neutral words and math equations, considering existing research demonstrating that lexical information triggers task conflict (Keha & Kalanthroff, 2023; Littman et al., 2019; Monsell et al., 2001) and the similarities between language and arithmetic abilities (Campbell, 1994; Noël et al., 1997; Rusconi et al., 2007), We expected RTs for the color naming of math equations to be at least equal to those for neutral words. Moreover, beyond the automatic stimulus-driven behavioral process that triggers task conflict and is induced by all types of math equations in the irrelevant dimension, our study aimed to explore additional memory retrieval processes by comparing different types of math equations. Firstly, drawing from the literature indicating heightened processing for larger equations (e.g., 6 × 9 = 54) compared to smaller ones (e.g., 2 × 3 = 6), we hypothesized that larger-size equations would yield longer RTs. Secondly, investigating the parity effect (Campbell et al., 2004; Lemaire & Reder, 1999), wherein different-parity incorrect answers (e.g., 2 × 3 = 7) hold an advantage in RT over same-parity incorrect answers (e.g., 2 × 3 = 8), we anticipated longer RTs for same-parity equations compared to different-parity equations. Finally, considering research demonstrating increased activity for correct compared to incorrect equations (Galfano et al., 2004), we hypothesized that correct and familiar equations would result in longer RTs than incorrect and less familiar equations.

## Method

### Participants

Fifty-six university students took part in the experiment in return for course credit or a small monetary reward (~6 USD). All participants had normal or corrected-to-normal vision, reported having no history of attention deficit or dyslexia, were native speakers of Hebrew, and were naive as to the purpose of the experiment. All participants signed up for the experiment via the university’s experiment system. One participant was excluded from the analysis due to low accuracy rates (>2.5 SDs below the group’s mean accuracy). Therefore, the analyzed sample included fifty-five participants (see Appendix B), 16 males, and 39 females, with a mean age of 22.96 (SD = 4.55). A power analysis using G*Power 3.1 (Faul et al., 2007) indicated that the current sample allowed for the examination of the one-way (trial-type) and of the two-way (size × parity or size × correctness) analyses of variance (ANOVAs) at a power >95% to test a small to medium effect size with a Type 1 error (α < 0.05). The effect sizes that were chosen for the analyses were based on previous studies that found small to medium effect sizes in color naming tasks that involve the processing of irrelevant neutral-words (Keha & Kalanthroff, 2023). Since there was a wide range between small to medium effect sizes, we chose the minimal effect size (Cohen’s *f of 0.2*) to ensure that the required sample was sufficient to examine our hypotheses.

### Stimuli and Materials

The experiment was designed and conducted using E-prime software version 3.0.3.80 (by Psychology Software Tools) and was administered online using the E-prime Go interface using a Windows operating system only. Participants were asked to respond to the ink color of the presented stimuli. All stimuli in the present experiment could appear in one of four colors: red, green, blue, or yellow. All 4 colors appeared equally. The experiment consisted of four different types of stimuli (1) math equations – 5 to 6-character long math equations (see next paragraph), (2) neutral-symbols – 4-character long neutral-symbols strings (e.g., ####), (3) same-number – 6 character long same-number strings (e.g., 111111), and (4) neutral-words – 4 letters words in Hebrew (e.g., בנין (building)). The complete list of stimuli can be found in Appendix A.

The math equations were multiplication equations (see Appendix A, Table 1) with results that differed on two dimensions: (1) result-size – ‘large’ equations, with a result that is higher than 30 (e.g., 6 × 7 = 42), or ‘small’ equations, with a result that is lower than 30 (e.g., 3 × 2 = 6). This classification of large-size and small-size equations was based on the median result size of the equations that were used; (b) correctness – ‘correct’ equations, with a correct result (e.g., 4 × 3 = 12), or an ‘incorrect’ equation, with incorrect results (e.g., 4 × 3 = 13). Incorrect equations differed on ‘parity’ and could have been either ‘incorrect – different-parity’, in which the parity of the result in the equation was different than the parity of the correct result (4 × 3 = 13), or ‘incorrect – same-parity’, in which the parity of the results in the equation was identical to the parity of the correct result (4 × 3 = 14). Note that the result of each incorrect equation could differ by ±1 or ±2 numbers from the correct answer. Therefore, we had six possible conditions of math equations (size (large vs. small) × correctness (correct vs. incorrect), and incorrect different-parity vs. incorrect same-parity), and each of these six conditions had 18 different equations. Overall, 108 math equations were created. The complete list of all math equations can be found in Appendix A (Table 1) including their presented frequencies and proportions (Table 3. All stimuli appeared at the center of a black screen in Courier’s new font. Stimuli measured approximately 0.5 cm in height and 1.5 cm in width.

### Procedure

The study was approved by the Hebrew University ethical committee (HUJI-2021-06011) and all participants signed an informed consent form before they participated in the study. The study was administered online. The experiment appeared in full-screen mode (the resolution of the experiment was adjusted for each computer to present all stimuli in the same size). Participants were asked to respond as quickly and as accurately as possible to the ink color of each stimulus, using only their dominant hand. Responses were made using four keys of the numeric keypad (1–4) and each key was assigned to a color: red (1), green (2), blue (3), and yellow (4).

The experiment started with a key-mapping practice block that contained 48 trials, in which participants were asked to manually respond to the ink color of a colored asterisk that could appear in one of the four possible colors. The asterisk appeared on the screen for 2,000ms or until response and was followed by a 500ms blank screen interval. Immediately after the mapping practice, participants completed a 96 trials practice block followed by two experimental blocks of 424 trials each. The practice and experimental blocks were identical and for both, each trial began with a 500ms white fixation plus sign, followed by the target stimulus, that appeared for 2,000ms or until keypress. Each trial ended with a 1,000ms blank screen interval. In the case of two consecutive incorrect responses, feedback was displayed to the participants to pay attention to the correct key-response mapping. The practice block contained 96 trials of which 32 were neutral-words and 64 were neutral-symbols trials. In each block, 42.5% of the trials (180 trials) were math equations trials, 42.5% were neutral-symbols trials (180 trials), 7.5% were same-number trials (32 trials), and 7.5% were neutral-words trials (32 trials). All trials were presented in a random order within each experimental block and with a different random order for each participant.

## Results

**Figure 1:**
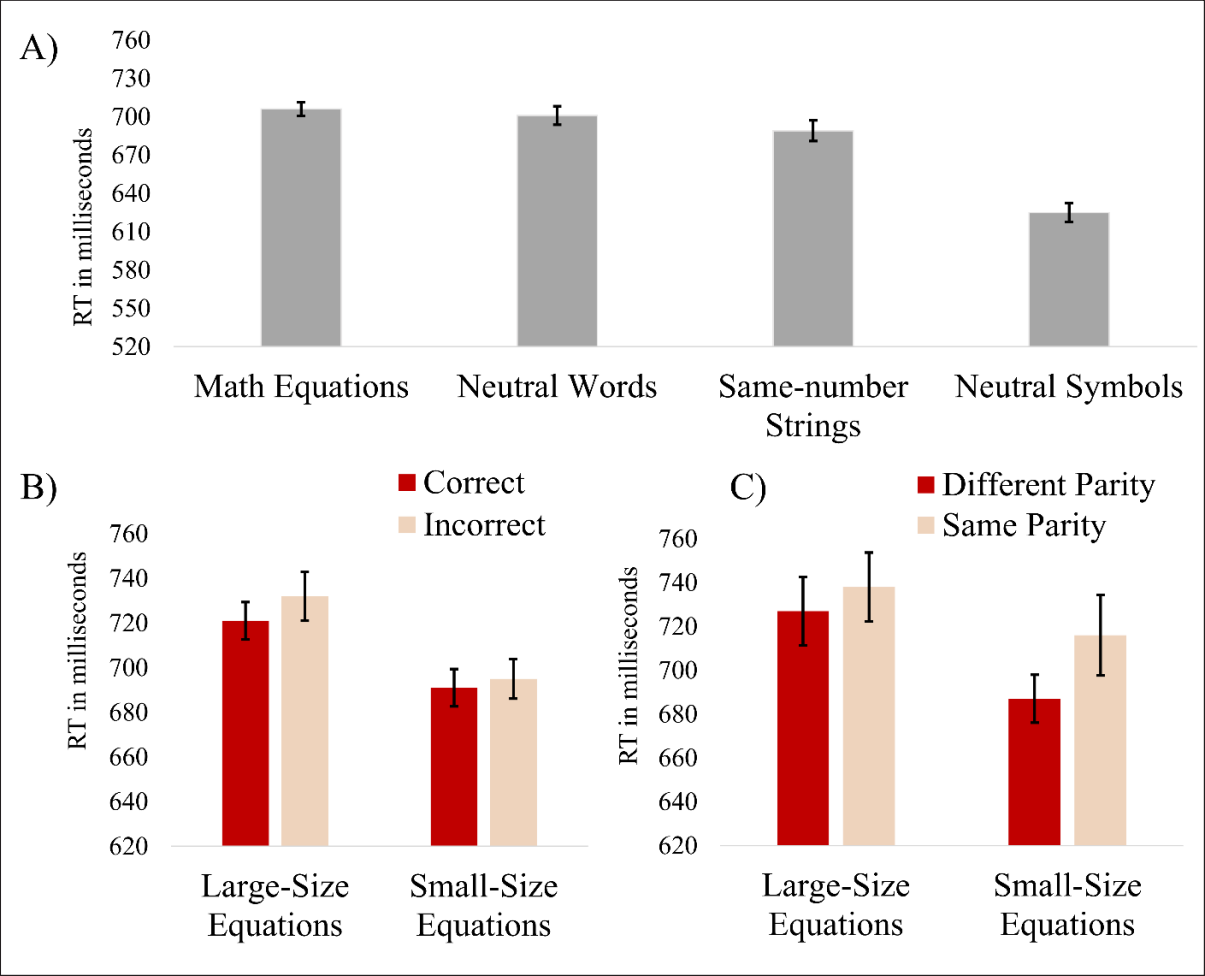
Mean reaction times of the different experiment conditions. *Note*. Panel A – mean reaction times (RTs) of the different stimulus type conditions. Panel B – RTs for incorrect (e.g., 4 × 6 = 23) and correct equations (e.g., 4 × 6 = 24) as a function of equations’ size. Panel C – RTs for incorrect equations as a function of equations’ size and parity of the incorrect results. Error bars represent one standard error from the mean (using Cousineau’s (2005) method to compute the error bars in within-subject designs).

Reaction time (RT) was calculated for each participant in each condition, for trials in which participants responded correctly to the ink color. Outlier trials (above or below 3 SDs from the mean of each participant in each condition) were excluded (an average of less than 2%). As in previous studies (LeFevre et al., 1996) we excluded equations that contained the number 5 in any of the equations (i.e., 5-operand problems) since these equations were found to be less intricate. We first tested whether math equations, in general, elicited task conflict by carrying out repeated measures one-way analysis of variance (ANOVA) on RT data with trial-type (math equation vs. neutral-symbols vs. same-number vs. neutral-words) as a within-subject factor (see Figure 1, Panel A). The effect of trial-type was significant, *F*(3, 162) = 55.61, *p* < .001, η^2^p = .507. Post-hoc comparisons, using a Benjamini-Hochberg correction, revealed that neutral-symbols were significantly faster compared to all other conditions, including math equations, *t*(54) = 13.57, *p* < .001, *d* = 1.83, same-number, *t*(54) = 7.93, *p* < .001, *d* = 1.07, and neutral-words, *t*(54) = 9.91, *p* < .001, *d* = 1.33. In addition, math equations were significantly slower than same-number trials, *t*(54) = 2.46, *p* =.026, *d* = 0.33, but did not differ from neutral-words, *t*(54) = 0.800, *p* = .427.

To further explore what kind of arithmetic processes were involved in the irrelevant dimension, we carried out two separate ANOVAs on RT data of correct and incorrect equations. First, a two-way repeated measures ANOVA was carried out on RT data with result-size (large vs. small) and correctness (correct vs. incorrect) as within-subject factors. Consistent with our hypotheses, a significant main effect was found for result-size, *F*(1, 54) = 22.27, *p* < .001, η^2^p = .292, with faster responses to the small result equations as compared to the large result equation (see Figure 1, panel B). However, there were neither main effect for correctness, *F*(1, 54) = 1.57, *p* = 215, nor an interaction between result-size and correctness, *F*(1, 54) = 0.29, *p* = .593. Next, a 2 × 2 two-way repeated measures ANOVA was carried out on RT data of incorrect math equations with result-size and parity (incorrect different-parity vs. incorrect same-parity) as within-subject factors. Results indicated, a main effect for result-size, *F*(1, 54) = 7.24, *p* = .009, η^2^p = .118, with faster responses to the small result equations compared to the large result equations, and a main effect for parity, *F*(1, 54) = 4.54, *p* = .038, η^2^p = .078, with a shorter reaction time for different-parity compared to same-parity equations. However, there was no significant result-size × parity interaction, *F*(1, 54) = 0.54, *p* = .464 (see Figure 1 Panel C).

Finally, we carried out an analysis of accuracy rates for color naming. Results yielded a significant effect for trial type, *F*(3, 152) = 9.65, *p* < .001, η^2^p = .152, driven only from a higher accuracy rate for the neutral-symbols condition compared to all other conditions (see Supplementary, Table 1), and a significant effect for parity equations such that responses to different-parity equations were more accurate as compared to responses to same-parity equations (see Supplementary, Table 2), *F*(1, 54) = 8.94, *p* = .004, η^2^p = .142. Other than that, accuracy rates were very high (M = 91.08, SD = 4.85) and no other effects or interactions were found.

## Discussion

In the present study, we had two main goals – the first was to provide evidence that math equations can be triggered in a stimulus-driven manner while performing a non-numerical color naming task. The second goal was to test which arithmetic processes are specifically triggered in the irrelevant dimension. To that end, we asked participants to name the color of different stimuli, including multiplication equations. Two different processes were examined – we first tested whether math equations that appeared as an irrelevant task at the irrelevant dimension triggered task conflict. We then tested whether these equations that activated task conflict, also triggered retrieved information based on the parity, size, or correctness of the equations. Our results demonstrated the existence of a stimulus-driven behavior that is triggered by the mere perception of math equations, which is in line with previous evidence showing that math equations can be processed automatically (Logan, 1990; Galfano et al., 2004). Specifically, math equations triggered more task conflict from the irrelevant dimension as compared to symbols and even to same-number strings and triggered a comparable task conflict to that triggered by neutral-words – a stimulus that is known to trigger task conflict by triggering the task of reading (Goldfarb & Henik, 2007; Keha & Kalanthroff, 2023). In addition, we found evidence for the notion that the stimulus-driven task that is triggered by these math equations is indeed related to mathematical processing: (a) large-size equations resulted in more task conflict compared to small-size equations, and (b) same-parity results triggered more task conflict compared to different-parity results. However, in contrast to other studies (Galfano et al., 2004) we did not observe any effect for the correctness of the equations, as responses to correct equations did not differ in accuracy when compared to responses to incorrect ones.

The prolonged color naming RTs observed for both math equations and neutral words, in comparison to single-digit numbers and neutral symbols, is consistent with prior findings indicating that more complex stimuli generally necessitate more time for color naming than simpler and less complex stimuli (Kim et al., 2008; Mitterer et al., 2003; Monsell et al., 2001). However, the effects of task complexity cannot fully explain stimulus-driven arithmetic processing, as evidenced by the observed result-size and parity effects, which consist of similar complexity levels. One could still argue, for instance, that larger equations were more character-intensive compared to smaller equations (or that different stimulus categories varied in character count), thus differing in complexity levels. Nevertheless, as demonstrated in previous research (Kinoshita et al., 2017), the length of a string does not notably influence color naming, specifically when employing a manual response paradigm.

That color-naming of large-size equations takes more time than small-size equations is consistent with several studies that show that larger-size equations are more difficult to solve than small-size equations (e.g., LeFevre & Liu, 1997; Penner-Wilger et al., 2002). Event-related potential (ERP) studies have shown that large-size equations elicit greater P2 activity, which likely represents higher-order perceptual and attentional processes, in frontal regions, compared to small-size equations (Kong et al., 1999). The result-size effect is also very similar to the frequency effect in other color naming tasks (Burt, 1994; 2002), which shows slower color naming for low-frequent words as compared to color naming of high-frequent words. In both cases, the harder it is to process the irrelevant dimension, the longer it takes to name the color. In that regard, it would also be hard to argue that small-size equations are more familiar than large-size equations since we did not observe any differences between familiar correct equations and non-familiar incorrect equations.

Our study also demonstrated that color naming of an incorrect equation becomes more challenging when the parity of the incorrect result matches the parity of the correct answer (e.g., 2 × 4 = 10 vs. 2 × 4 = 9). However, contrary to previous studies (Berch, 2005; Lemaire & Fayol, 1995), the parity effect was not larger in small-size equations. Moreover, Zbrodoff and Logan (1990) proposed that math equation verification involves assessing not just plausibility but also parity, which in turn can also signal if the answer is plausible or not. The observed parity effect mirrors the distance effect observed in the number-matching paradigm (Galfano et al., 2003), where longer RTs were noted when matching a presented number closer to the multiplication result of two numbers to be matched. Both tasks exhibited differences between incorrect equations, driven by a stimulus-driven process unrelated to the relevant task. Notably, in both studies, the less plausible answer prompted faster responses. However, in our study, the relevant task had no association with numerical information and yet arithmetic processes were triggered by the numerical irrelevant task.

Taken together, the current study’s results do not necessarily suggest that participants actively solved the equations. Individuals rarely engage in calculation when they have to decide whether an equation is correct or not, even when they are required to do so (Campbell & Fugelsang, 2001). However, the fact that we found an effect for result-size, and an effect for parity of the incorrect response, along with the fact that we found larger task conflict in equations than in numerical strings, strongly indicates that the conflict that was triggered by the irrelevant math equations occurred due to mathematical processing. Thus, we suggest that certain equations in addition to task conflict, elicit an equations’ retrieved information from memory, effectively amplifying engagement with the mathematical task. This does not necessarily imply that these equations possess a more inherently ‘equation-like’ quality, but rather that they prompt a more robust activation of task conflict. Moreover, our results also indicate that automatic mathematical processing induces more pronounced stimulus-driven behavior and consequently greater task conflict compared to simple number processing. This raises questions regarding the potential processes triggered by the presentation of same-number strings. The distinction observed between same-number strings and neutral symbols suggests the involvement of additional processes like counting or semantic processing of numbers, present in both math equations and same-number strings. Future studies should explore whether different tasks (in this case, different numerical tasks) induce or do not induce conflict, similar to studies noting differences in task conflict activation among various stimuli, depending on the task (Keha & Kalanthroff, 2023; Kinoshita et al., 2017).

The conflict that was triggered by the irrelevant equations was not only larger than the conflict triggered by numerical strings, but it was also comparable to the conflict triggered by actual words, which are known to trigger the obligatory task of reading. This is to say, that our results suggest that mathematical processing might be just as automatic as reading. In other words, we found that automatic numerical task activation is not limited to simple number or size perception (Dadon & Henik, 2017). Rather, our findings suggest that the system automatically activates complex arithmetic functions, even if these functions are irrelevant to one’s current goals. In contrast to words, math equations are not stimuli that we are exposed to on a daily base and yet we have demonstrated that they are automatically processed. The ability to automatically process arithmetic information implies that there is a fundamental inclination to act on mathematical equations. This fact raises an interesting question from a developmental perspective. Specifically, when exactly this automatic ability is developed and whether this ability can be a marker for individual differences in math in general. For example, it was found that children with developmental dyscalculia are not functionally processing numerical information in the irrelevant dimension and do not show the basic congruency effects between numerical information and physical size (Ashkenazi et al., 2009; Rubinsten & Henik, 2005). Thus, a reasonable hypothesis will be that developmental dyscalculia is also characterized by a lack of stimulus-driven engagement with mathematical equations. Future studies can test this hypothesis alongside the flip side of this coin – whether gifted children are characterized by increased stimulus-driven processing of mathematical equations.

In summary, the results of the current study demonstrate that automatic processing of mathematical equations takes place even when such equations appear in the irrelevant dimension (thus requiring no action). Our results not only show that math equations can be processed in a stimulus-driven fashion, but they also provide insight regarding what exact numerical processes are activated in the irrelevant dimension. Interestingly, the results suggest that mathematical processing might be just as automatic as word reading. Although we believe our results show that automatic mathematical processing is triggered by the equations, understanding the specific mathematical process that is automatically triggered awaits future studies.

## Data Accessibility Statement

Data for the present experiment is available at the OSF link attached in Appendix B. The data contain individual means of the different conditions in the present study.

## Additional File

The additional file for this article can be found as follows:

10.5334/joc.372.s1 Supplementary File. The Supplementary file includes the accuracy rates analysis.

## Appendices

### Appendix A

**Table 1:** Math equations that were used in the present study.

CORRECT SMALL	CORRECT LARGE	INCORRECT SAME-PARITY SMALL	INCORRECT SAME-PARITY LARGE	INCORRECT DIFFERENT-PARITY SMALL	INCORRECT DIFFERENT-PARITY LARGE

2 × 4 = 8	4 × 8 = 32	2 × 4 = 10	4 × 8 = 30	2 × 4 = 9	4 × 8 = 31

2 × 6 = 12	4 × 9 = 36	2 × 6 = 10	4 × 9 = 34	2 × 6 = 11	4 × 9 = 35

2 × 7 = 14	5 × 7 = 35	2 × 7 = 16	5 × 7 = 37	2 × 7 = 15	5 × 7 = 34

2 × 8 = 16	5 × 9 = 45	2 × 8 = 14	5 × 9 = 43	2 × 8 = 17	5 × 9 = 44

3 × 5 = 15	5 × 8 = 40	3 × 5 = 13	5 × 8 = 38	3 × 5 = 16	5 × 8 = 39

3 × 6 = 18	6 × 5 = 30	3 × 6 = 20	6 × 5 = 32	3 × 6 = 17	6 × 5 = 31

3 × 7 = 21	6 × 7 = 42	3 × 7 = 23	6 × 7 = 40	3 × 7 = 22	6 × 7 = 41

3 × 8 = 24	6 × 8 = 48	3 × 8 = 22	6 × 8 = 46	3 × 8 = 23	6 × 8 = 49

3 × 9 = 27	7 × 5 = 35	3 × 9 = 29	7 × 5 = 37	3 × 9 = 28	7 × 5 = 36

4 × 2 = 8	7 × 8 = 54	4 × 2 = 10	7 × 8 = 56	4 × 2 = 7	7 × 8 = 57

4 × 3 = 12	7 × 9 = 63	4 × 3 = 14	7 × 9 = 65	4 × 3 = 13	7 × 9 = 62

4 × 6 = 24	8 × 4 = 32	4 × 6 = 26	8 × 4 = 34	4 × 6 = 23	8 × 4 = 31

5 × 2 = 10	8 × 5 = 40	5 × 2 = 8	8 × 5 = 42	5 × 2 = 9	8 × 5 = 41

5 × 3 = 15	8 × 6 = 48	5 × 3 = 17	8 × 6 = 50	5 × 3 = 14	8 × 6 = 47

6 × 2 = 12	8 × 7 = 56	6 × 2 = 14	8 × 7 = 58	6 × 2 = 13	8 × 7 = 57

6 × 4 = 24	9 × 5 = 45	6 × 4 = 22	9 × 5 = 47	6 × 4 = 25	9 × 5 = 46

7 × 2 = 14	9 × 6 = 54	7 × 2 = 16	9 × 6 = 56	7 × 2 = 13	9 × 6 = 53

9 × 3 = 27	9 × 7 = 63	9 × 3 = 29	9 × 7 = 61	9 × 3 = 26	9 × 7 = 64

* All equations could randomly appear in one of 4 colors. Note that equations that included the number 5 were presented to participants but were excluded from the analysis. The color-to-equation pairing was random.

**Table 2:** Non-equation stimuli that were used in the present study.

SAME-NUMBER STRINGS	NEUTRAL-SYMBOLS	NEUTRAL-WORDS	PRESENTED COLOR

999999	^^^^	בורג Screw	Blue

111111	$$$$	מגמה Tendency	Green

444444	####	טופס Form	Yellow

888888	@@@@	בנין Building	Red

Note that the neutral-words appeared in Hebrew.

**Table 3:** Frequencies and proportions of the presented stimuli.

MATH EQUATIONS	OVERALL FREQUENCY	OVERALL PROPORTION	PROPORTION WITHIN MATH EQUATIONS

Correct Small	**60**	**7.08%**	**16.66%**

Correct Large	**60**	**7.08%**	**16.66%**

Incorrect Same-Parity Small	**60**	**7.08%**	**16.66%**

Incorrect Same-Parity Large	**60**	**7.08%**	**16.66%**

Incorrect Different-Parity Small	**60**	**7.08%**	**16.66%**

Incorrect Different-Parity Large	**60**	**7.08%**	**16.66%**

**NON-MATH EQUATIONS**			**PROPORTION WITHIN NON-MATH EQUATIONS**

Neutral-words	**64 (56.8)**	**7.5%**	**13.25%**

Same-number strings	**64 (57.4)**	**7.5%**	**13.25%**

Neutral-symbols	**360 (335)**	**42.5%**	**73.5%**

Note that these numbers represent the total number of stimuli that were presented and not the number of trials in a single block. In parentheses are the average amount of trials that were analyzed per participant for each stimulus-type condition after all the exclusions (extreme RTs, Incorrect responses, and non-five operand equations).

### Appendix B

Scientific data for the experiment can be accessed at the following OSF link: https://osf.io/6se8q/. DOI 10.17605/OSF.IO/6SE.
