# Supplementary material for: Evidence for Automatic, Stimulus Driven, Arithmetic Processing of Single-digit Multiplication Problems
Source: J Cogn. 2024 Jun 5;7(1):49. doi: 10.5334/joc.372 (PMC11160399; doi:10.5334/joc.372)
Supplement: Supplementary File. — The Supplementary file includes the accuracy rates analysis. [file joc-7-1-372-s1.zip › joc-372_keha-s1/Supplementary.docx]

**Supplementary**

**Table 1 – Accuracy Rates for the Different Trial Types**

| **The comparison** | **Effect** | **Statistical Line** |
| --- | --- | --- |
| Math Equations - Same-number Strings | 0.40% | t(54) = 0.58 , p = .562 |
| Math Equations – Neutral-Symbols | -2.82% | t(54) = 5.92 , p < .001 |
| Math Equations – Neutral-Words | -0.82% | t(54) = 1.32 , p = .229 |
| Same-number Strings – Neutral-Symbols | -3.22% | t(54) = 4.83 , p < .001 |
| Same-number Strings – Neutral-Words | -1.21% | t(54) = 1.52 , p = .199 |
| Neutral-Symbols – Neutral-Words | 2.00% | t(54) = 3.19 , p = .005 |

**Table 2 – Accuracy Rates for Math Equations Effects.**

| **Result-size** | **Small Size** | **Large Size** | **Difference** | **Statistical Line** |
| --- | --- | --- | --- | --- |
|  | 90.08% (6.38) | 88.92% (6.13) | 1.15% | t(54) = 1.94 , p = .06 |
| **Parity** | **Different-Parity** | **Same-Parity** |  | |
|  | 90.67% (6.60) | 88.54% (6.76) | 2.13% | t(54) = 2.99 , p = .004 |
| **Correctness** | **Incorrect** | **Correct** |  | |
|  | 89.99% (6.24) | 89.36% (6.05) | 0.63% | t(54) = 1.19 , p = .23 |
