# Supplementary material for: Evidence for Automatic, Stimulus Driven, Arithmetic Processing of Single-digit Multiplication Problems
Source: J Cogn. 2024 Jun 5;7(1):49. doi: 10.5334/joc.372 (PMC11160399; doi:10.5334/joc.372)
Supplement: Supplementary File. — The Supplementary file includes the accuracy rates analysis. [file joc-7-1-372-s1.zip › joc-372_keha-s1/Supplementary.pdf]

Supplementary

Table 1 – Accuracy Rates for the Different Trial Types

The comparison	***Effect***	***Statistical Line***
Math Equations - Same-number Strings	0.40%	t(54) = 0.58 , p = .562
Math Equations – Neutral-Symbols	-2.82%	t(54) = 5.92 , p < .001
Math Equations – Neutral-Words	-0.82%	t(54) = 1.32 , p = .229
Same-number Strings – Neutral-Symbols	-3.22%	t(54) = 4.83 , p < .001
Same-number Strings – Neutral-Words	-1.21%	t(54) = 1.52 , p = .199
Neutral-Symbols – Neutral-Words	2.00%	t(54) = 3.19 , p = .005

Table 2 – Accuracy Rates for Math Equations Effects.

**Result-size**	**Small Size**	**Large Size**	**Difference**	**Statistical Line**
	90.08% (6.38)	88.92% (6.13)	1.15%	t(54) = 1.94 , p = .06
**Parity**	**Different-Parity**	**Same-Parity**		
	90.67% (6.60)	88.54% (6.76)	2.13%	t(54) = 2.99 , p = .004
**Correctness**	**Incorrect**	**Correct**		
	89.99% (6.24)	89.36% (6.05)	0.63%	t(54) = 1.19 , p = .23
